# Differential Responses of Two Broccoli (*Brassica oleracea* L. var Italica) Cultivars to Salinity and Nutritional Quality Improvement

**DOI:** 10.1100/2012/291435

**Published:** 2012-07-31

**Authors:** Chokri Zaghdoud, Carlos Alcaraz-López, César Mota-Cadenas, María del Carmen Martínez-Ballesta, Diego A. Moreno, Ali Ferchichi, Micaela Carvajal

**Affiliations:** ^1^Laboratoire d'Aridoculture et Cultures Oasiennes, Institut des Régions Arides, Route de Djerba Km 22.5, Médenine 4119, Tunisia; ^2^Department of Plant Nutrition, Centro de Edafología y Biología Aplicada del Segura (CEBAS-CSIC), Campus Universitario de Espinardo, Ap. de Correos 164, 30100 Murcia, Spain; ^3^Department of Food Science and Technology, Centro de Edafología y Biología Aplicada del Segura (CEBAS-CSIC), Campus Universitario de Espinardo, Ap. de Correos 164, 30100 Murcia, Spain

## Abstract

The comparative responses of two broccoli cultivars (*Brassica oleracea* var. Italica, cv. Parthenon and cv. Naxos) to a 15 d exposure to different NaCl levels were investigated. Salinity led to increased concentrations of Na^+^ and Cl^−^ ions in both cultivars, a disruption of the endogenous minerals levels in the shoots and roots—that varied with the cultivar and salt concentration—and decreases in the osmotic potential (Ψ_*π*_), root hydraulic conductance (*L*_0_), and stomatal conductance (*G*_*s*_). The reduced biomass of Naxos at moderate NaCl indicates greater sensitivity to salinity, compared with Parthenon. Parthenon accumulated more soluble sugars, for osmotic adjustment, whereas Naxos accumulated proline, which gave the two cultivars differing nutritional characteristics. The total glucosinolates (GSLs) content was not affected by salinity in Parthenon while it decreased significantly in Naxos as a consequence of the decrease in the indole GSL. However, Naxos accumulated more aliphatic GSLs under salt stress than Parthenon, which confers on this cultivar a greater nutritional value when cultivated under salinity.These results suggest that, at distinct salinity levels, each broccoli cultivar adopts a specific strategy, indicating the crucial role of the genetic background on the organoleptic and nutritional properties that each cultivar acquires.

## 1. Introduction

 Salt accumulation in irrigated soils is one of the main factors that diminish crop productivity, since most of the plant species cultivated are not halophytic. Therefore, it is important to understand how plants respond and adapt to salinity. Salt tolerance in plants is a complex phenomenon, which depends on a number of interrelated factors based on morphological, biochemical, and physiological processes [1, 2].

 The first response of plants to salt stress is osmotic adjustment. Salinity-stressed plants tend to accumulate compatible solutes in their cytoplasm, to balance ions in the vacuole [3]. These compatible solutes are low-molecular-mass compounds that do not interfere with normal biochemical reactions. They include proline (Pro), glycine betaine [4], sugars [5], and polyols [6]. Generally, compatible solutes protect plants from stress through different processes, including contributions to the cellular water economy, detoxification of reactive oxygen species, protection of membrane integrity, and stabilisation of enzymes/proteins [7].

High external levels of NaCl reduce root hydraulic conductance (*L*_0_) [8] because of a toxic effect that has a negative influence on the concentration or functionality of aquaporins [9–11]. The decrease of the root *L*_0_ by salinity has been shown to lower the water flow from roots to shoots, even in osmotically adjusted plants [12, 13]. This decrease in water flow may lower the leaf water content, resulting in stomatal closure in order to maintain the water status [13, 14]. Salinity-provoked reduction of stomatal conductance (*G*_*s*_) has been reported in plants of *Phaseolus* [15], rice [16], amaranth [17], and mangrove [18]. These authors attributed the decreases of *G*_*s*_ and, in turn, of photosynthetic carbon assimilation, to the toxic effects of Na^+^ and Cl^−^.

Glucosinolates (GSLs) are sulphur-containing compounds characteristic of the Brassicaceae and are recognised as having human health-promoting effects related to sulphate assimilation [19]. It has been shown that the glucosinolate content can be altered by environmental factors, such as temperature and photoperiod [20], season [21] or sulphur fertilisation [22]. Under salt stress, GSLs have been observed to increase [23], suggesting that under low water potential they could be involved in osmotic adjustment and might be an adaptive component of salt tolerance [24]. However, more information is needed regarding the effect of external stress on GSL accumulation in plants. Phenolic compounds, to which are attributed health-promoting effects due to their antioxidant properties [19], have been found in high amounts in broccoli (*Brassica oleracea *var. Italica) [25]. Salt stress has been shown to induce disturbances in the secondary metabolic pathways, leading to increased accumulation of phenolic compounds [26].

Broccoli is a recognised health-promoting vegetable and one of the most important vegetables produced in the Southeast of Spain, under semiarid climatic conditions, and it is consumed frequently by people from both Western and Eastern cultures [27]. Broccoli plants are moderately salt tolerant [28], and the mechanism of tolerance has been studied by our group for many years.

Thus, the aim of this work was to compare the behaviours of two broccoli cultivars (Parthenon and Naxos) in relation to their osmotic adjustment to salt stress and variations of health-promoting compounds under salinity. For that, plant growth, water relations, proline, and soluble sugars as well as mineral content, glucosinolates, and phenolic compounds were determined. The effects of the saline treatments and cultivar factor were analysed individually.

## 2. Materials and Methods

### 2.1. Plant Growth and Experimental Design

Two broccoli cultivars (*Brassica oleracea* var. italica, cvs. Parthenon and Naxos) were used for the experiments. While Parthenon has been studied widely, Naxos is introduced here as a new cultivar highly tolerant of abiotic stress. The two studied varieties differ in their culture cycle and are the main varieties in the regional market.

Seeds of broccoli (cvs. Parthenon and Naxos, kindly provided by SAKATA Seeds Iberica) were prehydrated with deionised water and aerated continuously for 12 h. The seeds were then placed on trays with vermiculite as substrate before being placed in an incubator chamber at 28°C, in darkness. After 2 d, they were transferred to a controlled environment chamber with a 16 h light and 8 h dark cycle, with air temperatures of 25 and 20°C, respectively. The relative humidity (RH) was 60% (light period) and 80% (dark), and the photosynthetically active radiation (PAR) was 400 *μ*mol m^−2^ s^−1^, provided by a combination of fluorescent tubes (TLD36W/83, Philips, Hamburg, Germany and F36W/GRO, Sylvania, Danvers, MA, USA) and metal-halide lamps (HQI, T 400W; Osram, München, Germany).

After 3 d, the seedlings were placed in 15-L containers (10 plants per container) filled with continuously aerated Hoagland nutrient solution: KNO_3_ (3.0 mM), Ca(NO_3_)_2_ (2.0 mM), KH_2_PO_4_ (0.5 mM), MgSO_4_ (0.5 mM), H_3_BO_3_ (25.0 *μ*M), MnSO_4_ (2.0 *μ*M), ZnSO_4_ (2.0 *μ*M), CuSO_4_ (0.5 *μ*M), (NH_4_)_6_Mo_7_O_24_ (0.5 *μ*M), and Fe-EDTA (20.0 *μ*M). The solution was completely replaced every week. After 2 weeks of growth (when the plants were 17 d old), the plants were separated (5 per container with the same Hoagland nutrient solution) and treated with 0, 30, 60, or 90 mM NaCl. A Split-Plot design, with two factors (NaCl treatment, cultivar) and five replications, was used, to analyse each physiological variable. The salt treatments were applied to the nutrient solution by addition of 30 mM NaCl every hour until the final NaCl concentrations of 0, 30, 60, and 90 mM were reached, in order to avoid osmotic shock. After 15 d of NaCl treatment, the plants were harvested for analysis. Five replications of each treatment were used for the determinations.

### 2.2. Growth Parameters and Relative Water Content (RWC)

The total dry biomass was obtained after drying the fresh organs at 70°C until constant weight. The leaf area was measured every day after applying NaCl, using the method described by Paul et al. [29]. The relative water content (RWC) was calculated as % of water.

### 2.3. Analysis of Anions and Cations

The cation concentrations were determined in samples (ca 100 mg DW) of the oven-dried plant materials. The samples were digested, after HNO_3_-H_2_O_2_ (2 : 1) addition, in a microwave oven (CEM Mars Xpress, NC, USA). The concentrations of calcium (Ca^2+^), potassium (K^+^), magnesium (Mg^2+^), and sodium (Na^+^) were analysed by ICP spectrometry (Iris Intrepid II, Thermo Electron Corporation, Franklin, USA). 

The concentrations of chloride (Cl^−^), nitrate (NO_3_^−^), sulphate (SO_4_^2−^), and phosphate (PO_4_^3−^) were measured in a Dionex-D-100 ion chromatograph with an IonPac AS124-4 mm (10–32) column and an AG 14 (4 × 50 mm) guard column. The flow rate was adjusted to 1 mL min^−1^, with an eluent of 0.5 mM Na_2_CO_3_ and 0.5 mM NaHCO_3_.

### 2.4. Soluble Sugars and Proline Determination

The content of soluble sugars (SSs) in the leaf sap was determined according to Loewus [30]. The cell sap was collected from the most recent fully expanded leaves of the broccoli plants, which were frozen at −80°C and centrifuged at 5000 ×g for 5 min, at 4°C. The sap was then filtered through sep-pak C18 cartridges. An aliquot of the filtered sap was reacted with anthrone reagent (containing 5 mM anthrone and 70% (v/v) H_2_SO_4_) by heating in a water bath at 100°C for 10 min; and the absorbance of the coloured complex was measured at 650 nm using a spectrophotometer (Beckman DU-40).

The free Pro contents were determined according to the method of Bates et al. [31], with some modifications. The leaf sap used for SS determination was filtered again, through a 0.45 *μ*m pore-size filter. The reaction was initiated by combining 200 *μ*L of the sample, 250 *μ*L of ninhydrin reagent (containing 140 mM ninhydrin, 60 % (v/v) glacial acetic acid and 6 M phosphoric acid), 250 *μ*L of 99% (v/v) glacial acetic acid, and 400 *μ*L of distilled water; this mixture was incubated for 1 h at 100°C. The absorbance of the chromophore in toluene was measured at 515 nm in a spectrophotometer, using toluene as blank. A standard curve was prepared for each assay, using different dilutions of a 50 *μ*g/mL Pro stock solution.

### 2.5. Leaf Osmotic Potential

The most-recent fully-expanded leaves were put into Eppendorf tubes and frozen rapidly with liquid nitrogen. They were subsequently thawed and centrifuged to extract the cell sap.

The osmotic potential (Ψ_*π*_) of the leaf sap was calculated from the sap osmolarity, measured using an automatic, freezing-point-depression osmometer (Digital Osmometer, Roebling, Berlin), by the van't Hoff equation (Nobel 1991) [32]:

(1) $$\begin{array}{c} \Psi_{\pi }=nRT, \end{array}$$

where *n* is the osmotic concentration (mOsmol), *R* is the gas constant (0.083 L atm K^−1^ mol^−1^), and *T* is the ambient temperature (K).

### 2.6. Stomatal Conductance and Root Hydraulic Conductance

The adaxial stomatal conductance (*G*_*s*_) was measured every day for 15 d after applying NaCl, using a portable photosynthesis system (model LCA-4, ADC BioScientific Ltd., Hoddesdon, UK) and a PLC-4N leaf chamber (11.35 cm^2^), configured to an open system. The most-recent fully-expanded leaves were chosen for the analysis. The measurements were made in the middle of the photoperiod in order to obtain the highest values.

The root hydraulic conductance (*L*_0_) was measured by natural exudation. Three days before the measurements, plants of the different treatments were separated in 1 L containers, individually. For *L*_0_ measurements, the aerial parts of the plants were removed, leaving the base of the stem, which was sealed with silicone grease into a tapered plastic tube. The sap accumulated was collected in Eppendorf tubes. The roots and the Eppendorf tubes were weighed in a precision balance. The sap flow (*J*_*v*_) was expressed in mg g (root fresh weight)^−1^ h^−1^. The osmotic pressure difference (ΔΨ_*π*_) between the *J*_*v*_ and the corresponding nutrient solution was calculated according to their osmolarity values. The *L*_0_ was determined by the following equation:

(2) $$\begin{array}{c} L_{0}=\frac{J_{v}}{\Delta \Psi_{\pi }}\;\text{mg}\;\text{g}\;{\left(\text{root}\;\;\text{fresh}\;\;\text{weight}\right)}^{-1}\;{\text{h}}^{-1}\;{\text{MPa}}^{-1}. \end{array}$$

The measurements were made in the middle of the photoperiod, 15 days after applying NaCl.

### 2.7. Glucosinolate and Phenolic Compounds Determination

Glucosinolates and phenolic compounds were analysed according to the procedure described by Domínguez-Perles et al. [33] being expressed as mg per g of fresh weight and mg per 100 g of fresh weight, respectively. 

### 2.8. Statistics

The statistical analyses were performed using SPSS Release 18 for Windows. Significant differences between treatments for each cultivar were determined according to Tukey's test at *P* ≤ 0.05. Significant differences between cultivars were determined according to the Student's *t*-test at *P* ≤ 0.05, *P* ≤ 0.01, and *P* ≤ 0.001.

## 3. Results

The shoot and root dry weights (DWs) were decreased by salinity for both cultivars (Table 1). However, the decreases were more pronounced in plants of cv. Naxos than for Parthenon (*P *≤ 0.001) at 60 mM NaCl. Thus, at 60 mM NaCl, significant reductions were observed only for Naxos; compared with the control plants, shoot DW was reduced by about 17% and 43% for Parthenon and Naxos, respectively, and root DW by 21% and 34%, respectively. Similarly, at 90 mM NaCl, reductions in the shoot DW of 44% and 54% were observed for Parthenon and Naxos, respectively. The relative water content (RWC) of the shoots and roots of cv. Parthenon did not change with salinity, but a slight increase at moderate NaCl concentration was observed for Naxos.

In control plants, the leaf area was greater for cv. Parthenon than for Naxos (*P *≤ 0.05) (Table 1). However, whereas the decrease in the shoot DW was greater for Naxos, the leaf area reduction was more pronounced in cv. Parthenon. At 90 mM NaCl, the leaf area reduction compared to the control was 48% and 38%, for Parthenon and Naxos, respectively (Table 1).

The results of the analyses of mineral nutrients in the shoots and roots are shown in Table 2. In control plants, the K^+^ concentration was much higher than those of other ions. In both cultivars, the Na^+^ and Cl^−^ levels increased with increasing NaCl concentration and these ions accumulated mostly in the shoots. In the shoots, both cultivars showed a progressive decrease of the K^+^ level with increasing salt stress. Comparing the cultivars, significant differences were observed at 30 mM NaCl (*P* ≤ 0.001), with a greater reduction in K^+^ for cv. Naxos. The root K^+^ concentration decreased with increasing salinity, but only in Naxos were there significant differences among the NaCl treatments. Similar results occurred for Ca^2+^ in the shoots of both cultivars. Also, the Mg^2+^ concentrations were decreased in the shoots by salinity, for both cultivars, with higher values being maintained by cv. Naxos (*P *≤ 0.001) at 30 and 60 mM NaCl, in relation to cv. Parthenon. The NO_3_^−^ levels were also decreased by salt stress but, in contrast to K^+^, significant differences among the external NaCl concentrations were recorded only in the roots of Parthenon. The SO_4_^2−^ level increased only in the shoots of Parthenon at 30 mM NaCl and decreased in the roots of this cultivar with all salt treatments. However, for Naxos, SO_4_^2−^ decreased only at 90 mM NaCl, in both shoots and roots. In contrast to Parthenon, the PO_4_^3−^ levels in the roots of Naxos were unaffected by salinity.

**Figure 1:**
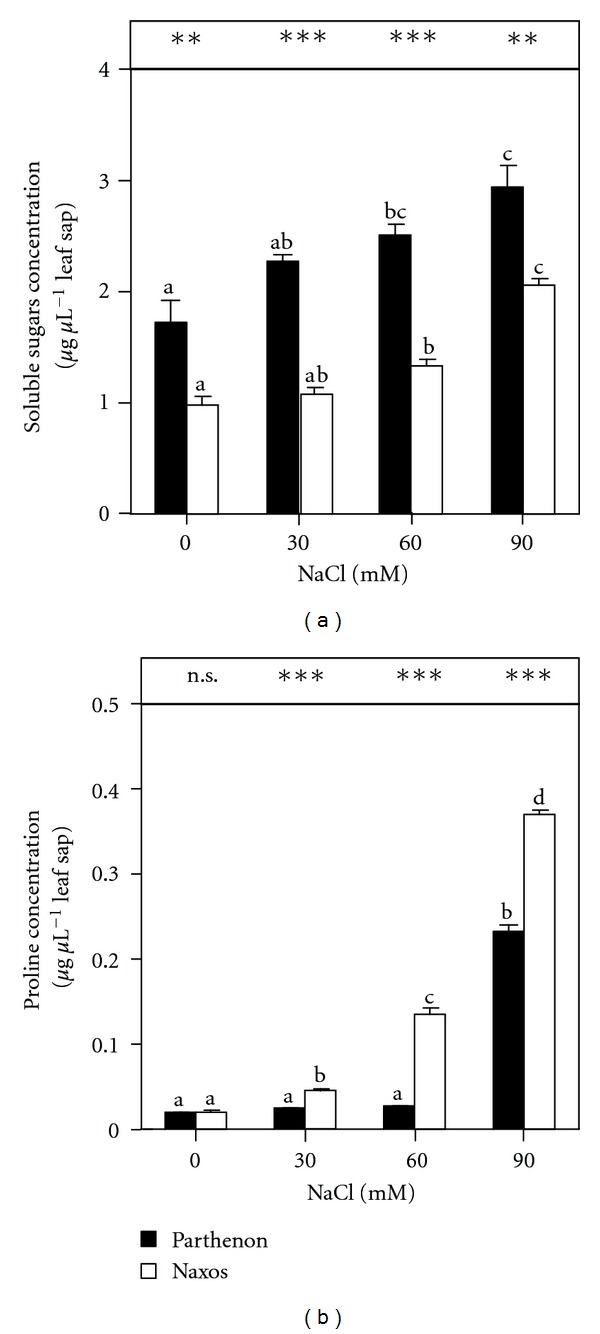
Leaf sap soluble sugars (a) and proline (b) concentrations of broccoli plants grown under saline conditions (0, 30, 60, or 90 mM NaCl) for 15 days (*n* = 5 ± SE). Column values with the same letters are not significantly different (*P* < 0.05 for soluble sugars and *P* < 0.01 for proline, Tukey's test). The significance of the difference between the two cultivars was given according to the Student's *t*-test, with *P < 0.05, **P < 0.01, ***P < 0.001; n.s: nonsignificant.

Plants of cv. Parthenon exhibited a higher SS concentration in their leaf sap than those of Naxos, under all salinity treatments (*P *≤ 0.001 at 30 and 60 mM NaCl and *P *≤ 0.01 at 90 mM NaCl) (Figure 1(a)). At 60 mM NaCl, the SSs were increased by 45% and 33% in the leaf sap of Parthenon and Naxos, respectively, compared to control plants; the increases reached 1.7-fold and 2-fold, respectively, at 90 mM NaCl. By contrast, in cv. Parthenon, an increased Pro concentration occurred only at the highest NaCl treatment (90 mM), whereas in cv. Naxos higher Pro accumulation was observed as salinity increased (Figure 1(b)). Leaf sap Pro levels were increased about 11-fold and 17-fold in 90 mM NaCl-treated Parthenon and Naxos plants, respectively, compared with control plants. Thus, leaf sap Pro accumulation differed significantly (*P *≤ 0.001) between cultivars for salt-treated plants but not under nonstressing conditions.

**Figure 2:**
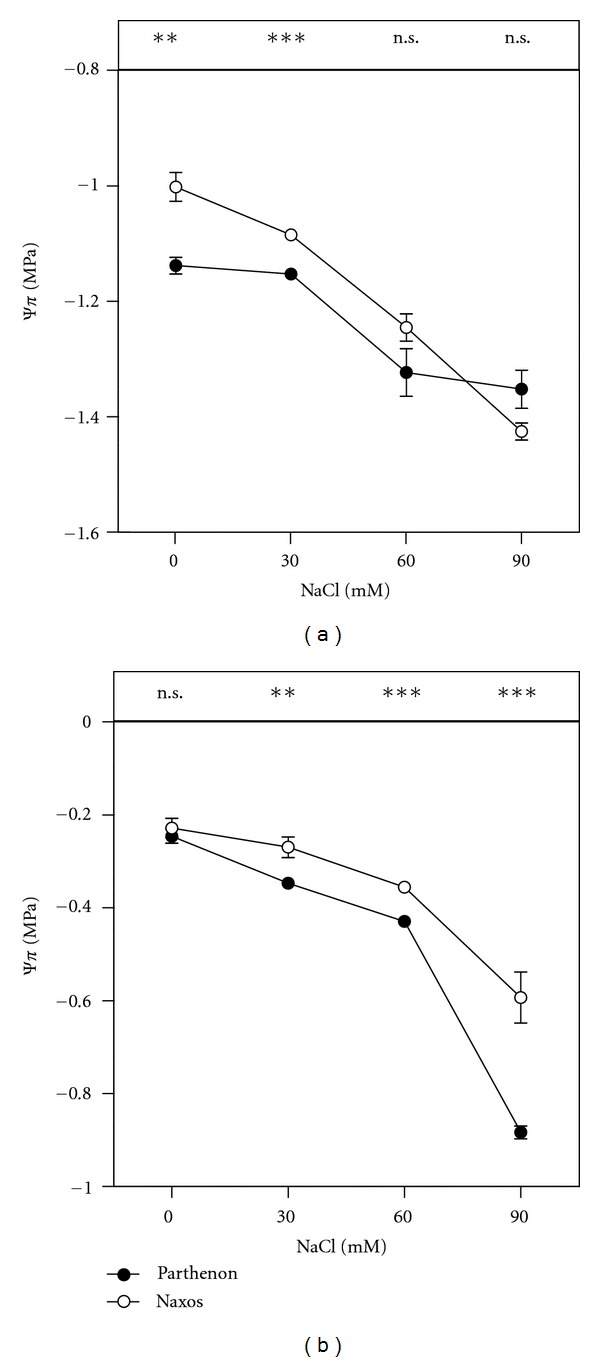
Osmotic potential (Ψ_*π*_) of leaf sap (a) and root xylem sap (b) of broccoli plants grown under saline conditions (0, 30, 60, or 90 mM NaCl) for 15 days (*n* = 5 ± SE). The significance of the difference between the two cultivars was given according to the Student's *t*-test, with *P < 0.05, **P < 0.01, ***P < 0.001; n.s: nonsignificant.

In Parthenon, the leaf sap Ψ_*π*_ was decreased only at the highest salt concentrations (60 and 90 mM NaCl), with respect to the control plants, whereas, in Naxos, it was reduced gradually by increasing salinity, the decrease reaching 42%, compared to the control value, at 90 mM NaCl (Figure 2(a)). However, at high salinity, the Ψ_*π*_ of the root xylem sap was decreased slightly in Naxos, compared to Parthenon (*P *≤ 0.001) (Figure 2(b)).

**Figure 3:**
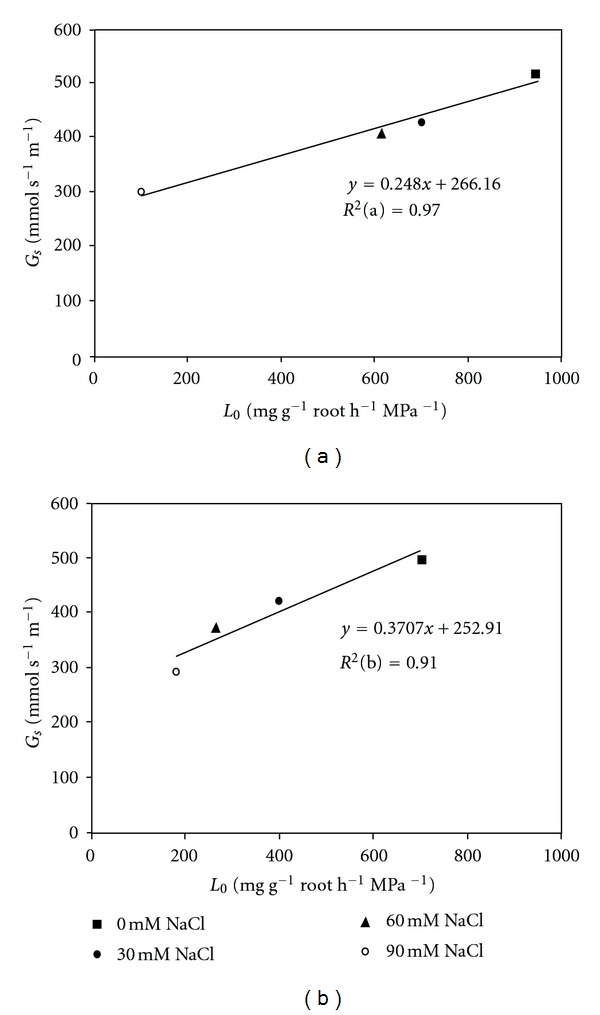
Correlation between stomatal conductance (*G*_*s*_) and root hydraulic conductance (*L*_0_) in cultivars Parthenon (a) and Naxos (b) grown under saline conditions (0, 30, 60, or 90 mM NaCl) for 15 days (*n* = 4 ± SE).

During the 15 days of treatment, salinity provoked gradual decreases in the *G*_*s*_ and *L*_0_ of the Parthenon and Naxos plants (Figure 3). However, the intensity of this decrease varied with the cultivar and the largest reduction in *L*_0_ at 90 mM NaCl was recorded in Parthenon. Our results show also a positive correlation between *L*_0_ and *G*_*s*_, in both cultivars.

**Figure 4:**
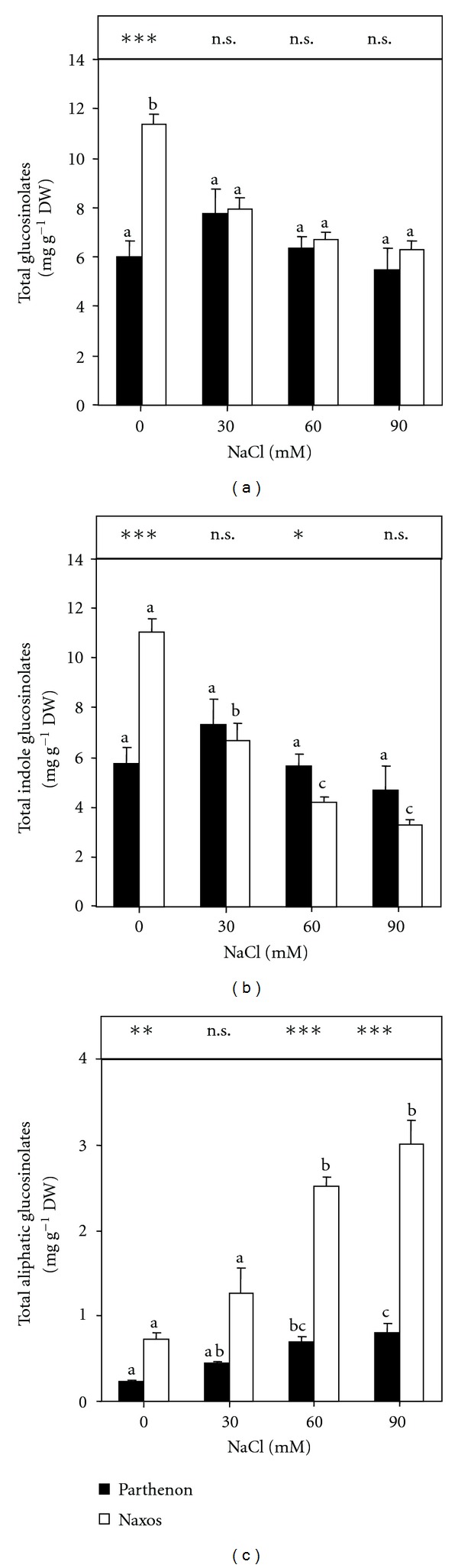
The concentrations of total glucosinolates (a), total indole glucosinolates (b), and total aliphatic glucosinolates (c) in the shoots of broccoli plants grown under saline conditions (0, 30, 60, or 90 mM NaCl) for 15 days (*n* = 5 ± SE). Column values with the same letters are not significantly different (*P* < 0.05, Tukey's test). The significance of the difference between the two cultivars was given according to the Student's *t*-test, with *P < 0.05, **P < 0.01, ***P < 0.001; n.s: nonsignificant.

The content of total GSL was expressed as the sum of the indole (glucobrassicin, neoglucobrassicin, 4-OH-glucobrassicin and 4-MeO-glucobrassicin) and aliphatic (glucoiberin, glucoraphanin, and glucoerucin) GSL analysed (Figure 4(a)). In control plants, the total GSL concentration was higher for Naxos than for Parthenon (*P* ≤ 0.001), a result of the higher neoglucobrassicin level in Naxos. The total GSL concentration in Naxos plants was decreased significantly by NaCl addition to the nutrient solution. This decrease reached 46.2% at 90 mM NaCl, compared to control plants and was the result of a greater decrease in the total indole GSL concentrations (Figure 4(b)), mainly glucobrassicin and neoglucobrassicin. In Parthenon, the total GSL concentration did not vary with the external NaCl concentration. In both cultivars, salinity induced an increase in the total aliphatic GSL (Figure 4(c)). At 60 and 90 mM NaCl, this increase was significant (*P* ≤ 0.001) for both cultivars and was related particularly to the increase in glucoerucin concentration. At 90 mM NaCl, the increase in total aliphatic GSL reached 4.18-fold and 3.34-fold in Naxos and Parthenon, respectively, compared to the control values (Figure 4(c)).

**Figure 5:**
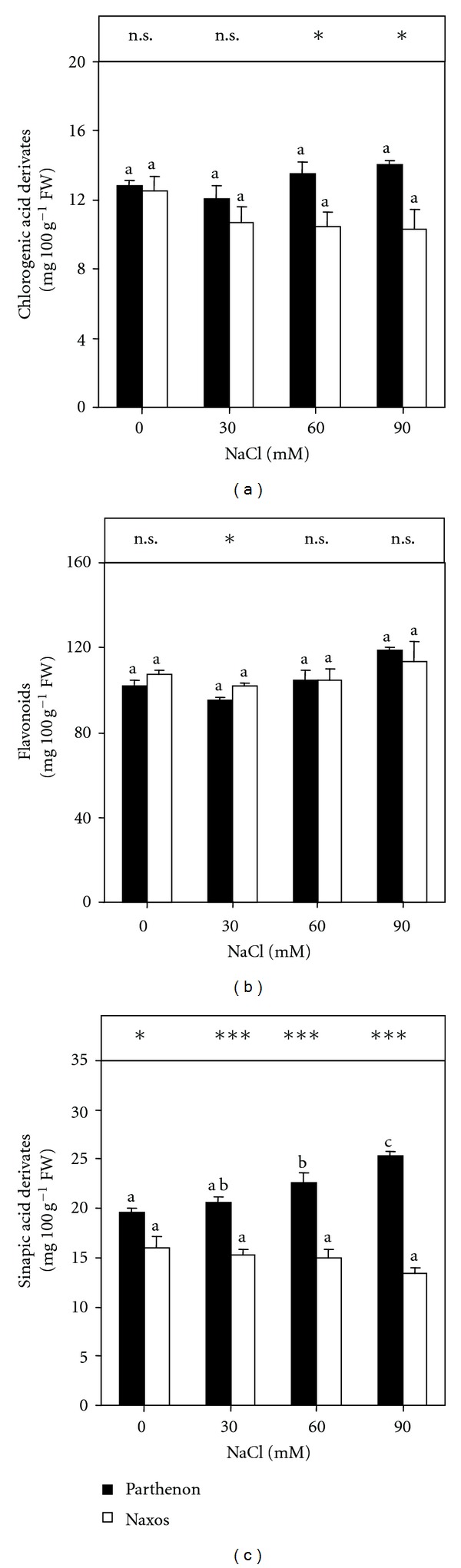
The concentrations of chlorogenic acid derivates (a), flavonoids (b), and sinapic acid derivates (c) in the shoots of broccoli plants grown under saline conditions (0, 30, 60, or 90 mM NaCl) for 15 days (*n* = 5 ± SE). Column values with the same letters are not significantly different (*P* < 0.05, Tukey's test). The significance of the difference between the two cultivars was given according to the Student's *t*-test, with *P < 0.05, **P < 0.01, ***P < 0.001; n.s: nonsignificant.

In this study, some natural antioxidants in broccoli are expressed as the content of phenolic compounds (chlorogenic, flavonoids, and sinapic acid derivatives). In the leaves of Naxos, there were no significant differences for any of the phenolic compounds analysed between control plants and salt-treated plants (Figure 5). However, in Parthenon, the levels of sinapic acid derivatives were enhanced significantly by increasing salinity, in comparison with cv. Naxos (*P *≤ 0.001) (Figure 5(c)).

## 4. Discussion and Conclusions

Salt stress induced a decrease in the shoot and root DWs of both broccoli cvs., which resulted in a constant root/shoot ratio and a reduction in the leaf area. However, these decreases in DW were more pronounced in cultivar Naxos than in Parthenon and the shoot biomass reduction for Naxos at moderate NaCl concentration indicates a greater sensitivity of this cultivar to salinity. In fact, shoot biomass has been shown as a trait for salt tolerance indication [34]. A similar decrease in the growth of broccoli plants under saline conditions was observed by López-Berenguer et al. [23, 28]. Leaf area decrease has been considered as the major cause of growth reduction, due to the decline in the photosynthetic area [35]. However, the greater decrease in the shoot DW of Naxos in relation to Parthenon, despite a greater leaf area reduction in Parthenon, could be explained by the reduction of the stem biomass for cv. Naxos.

Salinity induced a considerable accumulation of salt ions (Cl^−^ and Na^+^) in the plants, mainly Na^+^, whose level was higher in the shoots than in the roots. However, in the shoots, both cultivars maintained or increased their RWC under the salt treatments. Similar results were reported for different *Brassica* genotypes [36]. This stability of the RWC, despite the internal accumulation of salt ions, could reflect an inclusive response of broccoli plants to salinity, with salt being accumulated in the cells of the aerial organs, mainly in the vacuoles [37]. The vacuolar accumulation of salt ions under salinity leads to osmotic adjustment [38]. Thus, to achieve osmotic balance with the vacuole, the cytoplasm accumulates low-molecular-mass compounds, termed compatible solutes, because they do not interfere with normal biochemical reactions [3]; rather, they replace water in biochemical reactions. In accordance with this, there was a significant increase in the SS concentration in Parthenon plants even at the lowest external salt concentration (30 mM NaCl), whereas in Naxos a significant increase in these osmolytes was recorded only at 90 mM NaCl. Sugars act as osmotica and/or protect specific macromolecules and contribute to the stabilisation of membrane structures [39]. They also may protect cells during desiccation, by forming glasses [40]. By contrast, under saline conditions, higher accumulation of Pro was recorded in cv. Naxos. Besides osmotic adjustment, other roles have been proposed for Pro in osmotically stressed plant tissues: protection of plasma membrane integrity [41], a sink of energy or reducing power [42], a source of carbon and nitrogen [43], or a hydroxyl radical scavenger [44]. The differences in SS and Pro accumulation between cultivars Parthenon and Naxos under saline stress reflect the distinct strategies adopted by each cultivar to achieve osmotic adjustment. Zhu [45] reported that plants have to decrease their internal water potential in order to avoid the dehydration caused by salinity; this implies a decrease of the Ψ_*π*_, to maintain turgor and achieve osmotic adjustment. Anyway, the greater magnitude of the SS concentrations relative to Pro suggests the necessity for osmotic adjustment in Parthenon, which was not reflected in the leaf Ψ_*π*_; thus, accumulation of other osmolytes cannot be ruled out.

The *L*_0_ is known to decrease under high salinity [46, 47]. In the present study, salt stress led to a progressive reduction in the *L*_0_ values of both broccoli cultivars. The decrease in *L*_0_ can be attributed to the toxic effects of Na^+^ and Cl^−^, which reduce the passage of water through the plasma membrane. However, in other species, the *L*_0_ decrease was evaluated in terms of the osmotic effects of Na^+^ and Cl^−^ [48, 49]. The decrease in *L*_0_ in Parthenon and Naxos could also have been the result of a toxic effect of the high salt ion concentrations on root aquaporin functionality, since in previous work we showed a lack of correlation between *L*_0_ and the amount of PIP1 and PIP2 proteins under salt stress [50]. In fact, in Naxos, a larger increase in the amount of PIP2 proteins under the saline conditions imposed, compared with Parthenon, may have resulted in the rapid uptake of water into root cells, to dilute the NaCl that also entered the cells [51], therefore lowering the requirement for osmotic adjustment.

In addition, leaf *G*_*s*_ decreased in both broccoli cultivars with increasing external salinity, together with reductions in *L*_0_. Similarly, a strong correlation between *G*_*s*_ and *L*_0_ was shown in tomato plants under salt stress [52]. However, the higher slope value for Naxos could indicate a higher stomatal driving force for water transport through these plants under salinity.

Competition effects between different anions and different cations are known to occur in saline environments and they may be deleterious for plant growth [53]. In this work, the accumulation of Na^+^ in salt-treated broccoli induced significant decreases in the K^+^, NO_3_^−^, and Ca^2+^ concentrations in both shoots and roots, in agreement with previous results [54]. However, in cultivar Naxos, comparing the moderate- and high-salinity levels, the reductions of K^+^ in the roots and of Ca^2+^ and Mg^2+^ in the shoots differed significantly, whereas this level of significance was not observed in Parthenon, indicating differing degrees of sensitivity to moderate- and high-salinity for these two cultivars. In fact, the correlation between Na^+^ accumulation and shoot biomass reduction at different NaCl concentrations was higher in cv. Naxos (*r* ^2^ = 0.88) compared to Parthenon (*r* ^2^ = 0.77). The decreases in NO_3_^−^ concentrations caused by the NaCl treatments could have been due to inhibition of NO_3_^−^ uptake by Cl^−^ [55] and low NO_3_^−^ loading into the root xylem [56].

The GSLs are a category of secondary compounds found mainly in cruciferous plants such as broccoli, cauliflower, and cabbage [57]. Earlier work showed that environmental factors such as light [58], temperature [59], and heavy metals [60] alter the glucosinolate content and composition. At moderate salt stress, a tendency for the total leaf GSL to increase was observed in cv. Parthenon, accompanied by an increase in the SO_4_^2−^ concentration in the shoot. These findings agree with the recent results of Keling and Zhujun [61] for Pak choi plants, for which the total GSL concentration was increased at moderate salinity. Also, previous work showed that sulphur assimilation increased the GSL concentration under salinity [23]. The significant decrease (30% at 30 mM NaCl, around 40% at 60 and 90 mM NaCl) in the total GSL concentration in cv. Naxos was mainly the consequence of the decrease in the total indole GSL concentration. The differences between the two cultivars of broccoli with respect to their accumulation of SO_4_^2−^ and GSL under salt exposure suggest that glucosinolate synthesis under salinity depends on the genotype rather than the treatment.

The significant increases of the total aliphatic GSL in Parthenon (294% at 60 mM, 334% at 90 mM NaCl) and Naxos (348% at 60 mM, 418% at 90 mM NaCl) were mainly due to the higher accumulation of glucoerucin. Compensation for the decline in indole GSL by increased levels of aliphatic GSL may occur. In fact, the absence of aliphatic GSL in the *Arabidopsis thaliana* double mutant *myb28myb29 *led to an increase in the indole GSL [62]. Thus, under salt stress, aliphatic GSLs confer on this cultivar an increased nutritional value. Several products of the hydrolysis of methylsulphinyl aliphatic GSL, such as glucoraphanin (GR) and glucoiberin (GI), in broccoli are considered to reduce the risk of cancers. Sulphoraphane (derived from glucoraphanin) is the most potent, being a naturally occurring inducer of phase 2 enzymes that detoxify carcinogens [63].

Farnham et al. [26] and Scheuner et al. [64] reported that salinity induced an increase in the content of phenolic compounds, although the results referred mainly to the edible parts (florets, inflorescences). Conversely, a number of reports describe how phenolic compounds may decrease as a consequence of long-term plant exposure to NaCl treatments [65]. Therefore, the way in which abiotic stress factors may influence the content of bioactive phytochemicals of plant material is not absolutely clear. These divergences in the response to stressing factors indicate the importance of the genetic background in the response of broccoli to environmental factors, as in this paper, where the distinct secondary metabolic responses of cultivars Naxos and Parthenon under salt stress were reflected in the levels of both GSL and sinapic acid derivates.

In conclusion, cultivars Parthenon and Naxos employed differing strategies for growth under moderate and high salt stress, indicating the importance of the genetic background as a modifying factor. Naxos seems to be more sensitive, since its biomass reduction due to NaCl exposure was greater than that of Parthenon. The effect of salinity on endogenous mineral levels varied with the cultivar, organ, and NaCl concentration. In addition, to achieve osmotic adjustment, Parthenon accumulated more SS whereas Naxos accumulated more Pro, showing that the determinants of the response to salinity in broccoli, regarding osmotic adjustment, are genotype dependent and vary in both the ionic and osmotic phases of salt stress. The effect of salinity on the levels of bioactive compounds differed between the cultivars and conferred on them a greater nutritional value, especially for cv. Naxos due to increases in its aliphatic glucosinolates. Therefore, salt-affected lands could be suitable for broccoli cultivation, in order to obtain increased production of phytochemicals in this crop.

## Figures and Tables

**Table 1:** Dry weight (DW), relative water content (RWC), and leaf area of Parthenon and Naxos cultivars after different salt treatments for 15 days. Values are means ± standard errors (*n* = 5). Means followed by different letters are significantly different according to Tukey's test at *P* < 0.05. The significance of the difference between the two cultivars is given according to the Student's *t*-test, with ^∗^ *P* < 0.05, ^∗∗^ *P* < 0.01, ^∗∗∗^ *P* < 0.001; n.s: non-significant.

Cultivar	NaCl (mM)	DW (g plant^−1^)		Root : shoot	RWC (%)		Third leaf area
		Shoot	Root	ratio	Shoot	Root	(cm)
Parthenon	0	3.87 ± 0.23 a	0.55 ± 0.03 a	0.14 a	90.19 ± 0.36 a	91.88 ± 0.24 a	161.02 ± 8.01 a
	30	3.70 ± 0.32 a	0.54 ± 0.03 a	0.15 a	90.99 ± 0.12 a	92.05 ± 0.13 a	137.27 ± 11.14 ab
	60	3.19 ± 0.12 a	0.43 ± 0.03 ab	0.14 a	90.90 ± 0.08 a	92.14 ± 0.16 a	113.03 ± 2.60 bc
	90	2.17 ± 0.20 b	0.31 ± 0.02 b	0.15 a	90.68 ± 0.14 a	91.66 ± 0.17 a	83.84 ± 5.25 c
Naxos	0	3.07 ± 0.19 a	0.49 ± 0.05 a	0.16 a	90.40 ± 0.18 a	91.49 ± 0.27 ab	137.50 ± 2.43 a
	30	2.78 ± 0.14 a	0.47 ± 0.02 a	0.17 a	91.43 ± 0.10 ab	92.06 ± 0.34 b	134.74 ± 8.12 a
	60	1.74 ± 0.14 b	0.32 ± 0.02 b	0.19 a	92.21 ± 0.17 b	91.88 ± 0.25 ab	105.09 ± 4.16 b
	90	1.41 ± 0.14 b	0.26 ± 0.03 b	0.19 a	91.48 ± 0.10 ab	90.97 ± 0.38 a	85.17 ± 5.54 b

Significance between the two cultivars	0	^ ∗^	n.s.	n.s.	n.s.	n.s.	^ ∗^
	30	^ ∗^	n.s.	n.s.	^ ∗^	n.s.	n.s.
	60	^ ∗∗∗^	^ ∗^	^ ∗^	n.s.	n.s.	n.s.
	90	^ ∗^	n.s.	n.s.	^ ∗∗^	n.s.	n.s.

**Table 2:** Effects of salt stress on ion levels in the shoots and roots of cultivars Parthenon and Naxos treated with different concentrations of NaCl for 15 days. Values are means ± standard errors (*n* = 5). Means followed by different letters are significantly different according to Tukey's test at *P* < 0.05. The significance of the difference between the two cultivars is given according to the Student's *t*-test, with ^∗^ *P* < 0.05, ^∗∗^ *P* < 0.01, ^∗∗∗^ *P* < 0.001; n.s: non-significant.

Plant part	Cultivar	NaCl (mM)	Cations (mmol kg DW^−1^)				Anions (mmol kg DW^−1^)			
			Na^+^	K^+^	Ca^2+^	Mg^2+^	Cl^−^	NO_3_^−^	PO_4_^3−^	SO_4_^2−^
Shoots										
	Parthenon	0	4 ± 0.15 a	1292 ± 19.81 a	902 ± 14.50 a	143 ± 4.08 a	11.61 ± 0.69 a	86.32 ± 1.17 a	5.05 ± 0.33 a	14.39 ± 0.29 b
		30	901 ± 25.13 b	920 ± 11.26 b	727 ± 11.20 b	121 ± 2.12 b	58.58 ± 3.62 b	74.56 ± 1.28 b	6.51 ± 0.06 b	16.18 ± 0.38 c
		60	1438 ± 16.94 c	663 ± 21.41 c	645 ± 11.29 c	109 ± 1.81 b	84.64 ± 4.11 c	60.67 ± 0.42 c	6.53 ± 0.12 b	14.05 ± 0.06 b
		90	1878 ± 73.24 d	540 ± 20.86 d	588 ± 26.16 c	112 ± 5.30 b	145.26 ± 10.90 d	48.44 ± 3.40 d	6.34 ± 0.34 b	10.43 ± 0.51 a
	Naxos	0	19 ± 2.53 a	1290 ± 32.74 a	884 ± 11.51 a	159 ± 2.28 a	10.38 ± 0.67 a	76.22 ± 5.01 a	2.84 ± 0.65 a	12.56 ± 0.74 a
		30	1023 ± 50.65 b	795 ± 17.81 b	743 ± 6.21 b	139 ± 1.74 b	48.05 ± 6.37 b	57.21 ± 1.90 b	4.37 ± 0.20 b	11.66 ± 0.47 a
		60	1536 ± 43.54 c	669 ± 8.10 c	696 ± 12.00 c	140 ± 2.46 b	68.37 ± 2.03 c	50.47 ± 3.90 bc	5.07 ± 0.22 b	11.27 ± 0.08 ab
		90	2120 ± 86.19 d	546 ± 25.90 d	557 ± 10.08 d	128 ± 1.61 c	154.83 ± 5.64 d	43.46 ± 1.17 c	5.08 ± 0.19 b	9.23 ± 0.80 b
		0	^ ∗∗^	n.s.	n.s.	^ ∗^	n.s.	n.s.	^ ∗^	n.s.
	Significance between the two	30	n.s.	^ ∗∗∗^	n.s.	^ ∗∗∗^	n.s.	^ ∗∗∗^	^ ∗∗∗^	^ ∗∗∗^
	cultivars for the shoot ion composition	60	n.s.	n.s.	^ ∗^	^ ∗∗∗^	^ ∗∗^	n.s.	^ ∗∗^	^ ∗∗∗^
		90	n.s.	n.s.	n.s.	^ ∗^	n.s.	n.s.	^ ∗^	n.s.

Roots										
	Parthenon	0	8 ± 1.10 a	1295 ± 79.35 a	255 ± 26.65 a	99 ± 6.96 a	6.75 ± 2.01 a	48.72 ± 4.73 a	9.75 ± 0.15 a	6.45 ± 0.28 a
		30	388 ± 31.49 b	995 ± 77.89 b	144 ± 17.33 b	85 ± 5.86 a	31.55 ± 1.35 b	28.98 ± 2.47 b	5.93 ± 0.17 b	5.14 ± 0.25 b
		60	592 ± 32.52 c	919 ± 60.36 b	94 ± 2.41 b	88 ± 4.19 a	44.42 ± 2.44 c	19.95 ± 1.07 bc	6.09 ± 0.17 b	4.95 ± 0.21 b
		90	786 ± 35.21 d	756 ± 60.72 b	88 ± 3.36 b	84 ± 3.93 a	61.73 ± 2.63 d	14.90 ± 3.00 c	5.60 ± 0.40 b	4.62 ± 0.38 b
	Naxos	0	26 ± 2.09 a	1376 ± 10.07 a	253 ± 43.70 a	96 ± 2.80 a	7.72 ± 2.44 a	31.44 ± 4.06 a	6.67 ± 0.30 a	5.18 ± 0.21 a
		30	418 ± 18.45 b	1162 ± 25.48 b	176 ± 24.39 ab	96 ± 1.29 a	42.74 ± 2.57 b	23.13 ± 1.64 ab	5.93 ± 0.25 a	4.50 ± 0.14 a
		60	616 ± 30.70 c	1055 ± 51.20 bc	111 ± 3.19 b	95 ± 4.51 a	47.75 ± 3.89 b	22.22 ± 1.50 ab	5.50 ± 0.29 a	4.51 ± 0.12 a
		90	881 ± 38.22 d	991 ± 24.82 c	98 ± 7.80 b	105 ± 2.49 a	66.26 ± 3.52 c	17.59 ± 2.26 b	4.69 ± 0.85 a	3.38 ± 0.45 b
		0	^ ∗∗∗^	n.s.	n.s.	n.s.	n.s.	^ ∗^	^ ∗∗∗^	^ ∗∗^
	Significance between the two	30	n.s.	n.s.	n.s.	n.s.	^ ∗∗^	n.s.	n.s.	n.s.
	cultivars for the root ion composition	60	n.s.	n.s.	^ ∗∗^	n.s.	n.s.	n.s.	n.s.	n.s.
		90	n.s.	^ ∗^	n.s.	^ ∗∗∗^	n.s.	n.s.	n.s.	n.s.
